# An innovative oncoplastic technique for immediate small to medium volume breast reconstruction in lower inner quadrant cancer: The Zhuo-technique

**DOI:** 10.1016/j.amsu.2021.102576

**Published:** 2021-07-17

**Authors:** Wenjie Shi, Luz Angela Torres-de la Roche, Henning Ritter, Jie Dong, Jia-Jia Zeng, Yi-Cheng Jiang, Rui Zhuo, Rudy Leon De Wilde

**Affiliations:** a University Hospital for Gynecology, Pius-Hospital, University Medicine Oldenburg, 26121, Oldenburg, Germany; b Department of Breast Surgery, EUSOMA Certified Breast Center, Guilin TCM Hospital of China, Guilin, 541002, Guang Xi, PR China; c Director Sino-European Brest Care Nurse School in Guilin, PR China

**Keywords:** Oncoplastic approach, Breast cancer, Reconstructive surgery, Zhuo-technique

## Abstract

**Background:**

Insufficient glandular tissue in the lower quadrant of the breast is the main source of difficulty in repairing defects after oncoplastic surgery. Especially in small to medium sized breasts, this issue is more common. Here, we describe a novel oncoplastic approach that could help to solve this problem.

**Materials and methods:**

Retrospective analysis of breast cancer patients with tumors in the lower inner quadrants, who underwent Zhuo's technique between January 2017 and August 2019. Aesthetic outcomes were evaluated in terms of the Paris Breast Center's 5-point scale. The work was reported according to the STROCSS criteria.

**Results:**

Nine patients (mean age 54 years) with small to medium volume breast received Zhuo's oncoplastic technique after tumor excision. The mean size tumor was 18.0 mm. The median follow-up time was 27.0 months. Sentinel lymph node biopsy results for all patients were negative. None of the patients had local recurrences or metastases and postoperative complications were not observed. Seven patients (77.8%) achieved aesthetic scores of 5 and two patients (22.2%) achieved 4 points.

**Conclusions:**

Zhuo's oncoplastic technique could provide a favorable and flexible surgical approach for small to medium volume breast with tumors of the lower inner quadrant with a low risk of recurrence and good aesthetic results.

**Protocol register:**

Chinese clinical trial register No. ChiCTR2100043484.

## Highlights

• Defects after oncoplastic surgery is complicated by a lack of glandular tissue specially in case of small volume breasts.
• The Zhuo-technique generates a flexible pedicle flap from the upper abdomen to fill the defect area.
• The Zhuo-technique allows sufficient tissue transposition improving the postsurgical aesthetic appearance.

## 1 Introduction

Oncoplastic breast surgery (OPBS) combines tumor resection and breast reconstruction techniques. It plays an important role in the treatment of breast cancer. Compared with traditional breast conservative surgery (BCS) and mastectomy, OPBS aims to preserve the aesthetics and, thus, maintain the patient's positive body image to the greatest extent while allowing for complete tumor resection [1,2].

The widespread promotion of OPBS approaches has greatly improved the clinical benefits of breast cancer patients [3]. Many patients who may require breast tissue excision are thus exempted from mastectomy, thereby maintaining a better local appearance of the breast and improving their quality of life [4]. However, in our clinical practice, we have found that for Asian women with small to medium volume breasts, although the total volume of resection is small, it accounts for a large portion of the breast, and, therefore, deformities commonly occur. This is especially the case when the tumor is located in the lower inner quadrant (LIQ), which is a high-risk of cancer area [5]. The OPBS classic inverted T technique and simple glandular tissue transfer technique are often unable to achieve acceptable aesthetic results. At present, the most common solution is to use latissimus dorsi (LD) flap or immediate fat filling to repair these defects [6,7]. Though these methods may achieve good aesthetic results, the flap incisions can lead to postoperative shoulder dysfunction and eventually subsequent implant surgery, thereby reducing patient satisfaction. Therefore, an innovative, convenient, and efficient surgical technique is urgently needed to solve this clinical problem.

In the present study, we analyzed the results of a small cohort of breast cancer patients with tumors in the LIQ who underwent a novel surgical approach, Zhuo's oncoplastic technique. This surgical approach provides a flexible pedicle flap from the upper abdomen, close to inframammary fold (IMF), to fill the defect area; consequently, this technique can increase the chance of achieving good aesthetic outcomes after tumor resection.

## 2 Methods

### 2.1 Data resource

We used dataBreast app (https://app.databreastchina.cn/) to download the data of patients who underwent breast conserving surgery in the EUSOMA Certified Breast Center of Gui Lin TCM Hospital of China between January 2017 and August 2019. Follow-up time was limited to August 2020. Patients whose tumors were located in the lower inner quadrants and who received Zhuo's technique were included. Candidates' data had to include complete basic demographic characteristics, accurate pathological results, detailed treatment plans, and follow-up information. The protocol was approved by the Institutional Review Ethics Committee (GTCMH-2020-100; 2020.09.01) and was registered at Chinese clinical trial register, No. ChiCTR2100043484 (http://www.chictr.org.cn/showproj.aspx?proj=122030). The study was conducted according to the Declaration of Helsinki principles. The work has been reported according to the STROCSS criteria [8].

### **2.2** Zhuo's oncoplastic technique

The new technique for tumor resection and repair of the surgical defect in small to medium volume breasts with tumors in LIQ is performed in several steps:

#### 2.2.1 First step, tumor resection

**Fig. 1:**
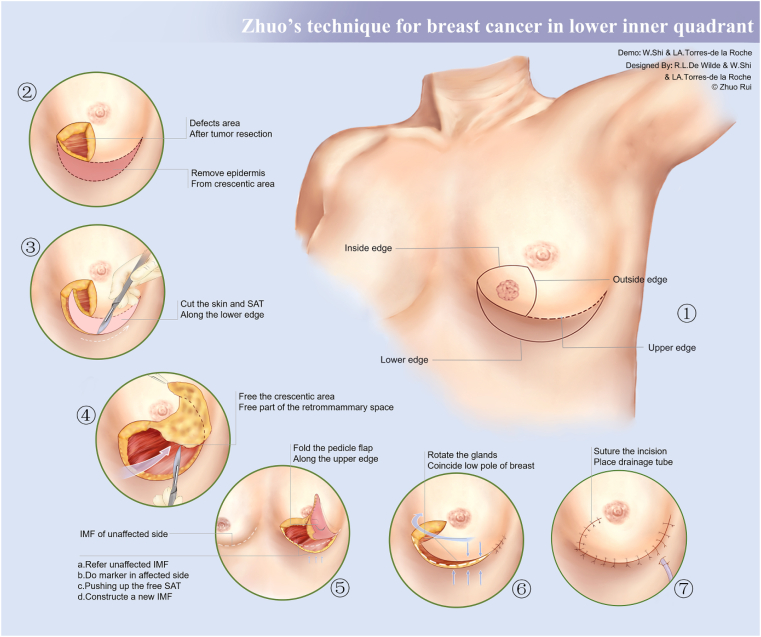
A demo figure for Zhuo's approach step by step.
b) Next, using the nipple as the apex, two skin incisions are made, medially and laterally respectively, from the tumor forming the inside and outside edges of the approach.
c) The patient is then asked to move to a sitting position to push the abdominal subcutaneous adipose tissue (SAT) close to the IMF of the affected side, which should be parallel to the IMF of the unaffected breast.
d) Subsequently, a crescent-shaped mark is made along the lower edge of the abdominal SAT that is pushed up. Once the abdominal SAT has been allowed to return to its normal position, the crescent-shaped mark will delineate the lower edge of the surgical approach.
e) The upper edge of the surgical incision is divided into two parts. The first part is a fan-shaped arc designed for tumor removal. The second part is a flexible extension of the pedicle flap, which can be modified according to the size of the residual cavity after tumor resection,
f) The IMF level of the healthy breast should be marked as a reference (Fig. 1.1).
g) The tumor should be completely resected and rapid freezing biopsy should be performed to determine whether the surgical margins are free of tumor. Patients should undergo sentinel lymph node (SLN) biopsy.

a) With the patient lying in the supine position, the tumor is located using ultrasound and marked with a circle (Fig. 1).

#### 2.2.2 Second step, defect repair

After the tumor has been resected, a cavity remains. A pedicle flap from the upper abdomen, closing to IMF, is used to fill the defect:

a) Remove the epidermis from crescentic area of the upper abdomen, cut the skin and SAT along the long side of the crescent (lower edge of approach) (Fig. 1.3),
b) Then, cutting along the lower edge, free the crescentic area completely and part of the retromammary space (Fig. 1.4),
c) Use the IMF of the healthy side as a reference and methylene blue to symmetrically delineate the new IMF on the affected side,
d) Free the SAT downward, along the long arc of the crescent incision, pushing up the free SAT, where the long arc of the crescent incision coincides with the methylene blue marking point, fix it and a new IMF is constructed,
e) Fold the pedicle flap along the upper edge of the incision and fix it with the subcutaneous fat layer to form a new lower pole of the breast. If there is tension in the closure, loosen the upper edge of the incision (Fig. 1.5),
f) Rotate the new lower pole of the breast inside. The inner and outer edges of the fan-shaped incision will automatically align, the new lower pole and the new IMF will also coincide (Fig. 1.6),
g) Suture the incision and place the drainage tube. Once a tension-free closure has been achieved, the operation is completed (Fig. 1.7).

### 2.3 Cosmetic evaluation

Immediately after the operation, the patient's breast shape should be evaluated by the surgical team. The long-term effects of the breast shape after the operations included in this study were evaluated by no less than three professional breast surgeons. The aesthetic standards were assessed using the 5-point scoring method, developed by the Paris Breast Center [9]. On this scale, points correlate to overall aesthetic results, with a score of one to five representing patient satisfaction from low to high (one point means bad aesthetic results, a score of five means excellent). These scoring standards are based on three indicators: 1) breast form: symmetry, retraction and deformation 2) nipple-areola complex (NAC) positions: perfect position; good position; minimal deviation of NAC toward the operated quadrant; deviation of NAC and 3) action required: no further action; aesthetic sequelae of conservative treatment ASCT type 1, requiring contralateral symmetrization; ASCT type 2 requiring ipsilateral reoperation; ASCT type 3 mastectomy.

## 3 Results

**Fig. 2:**
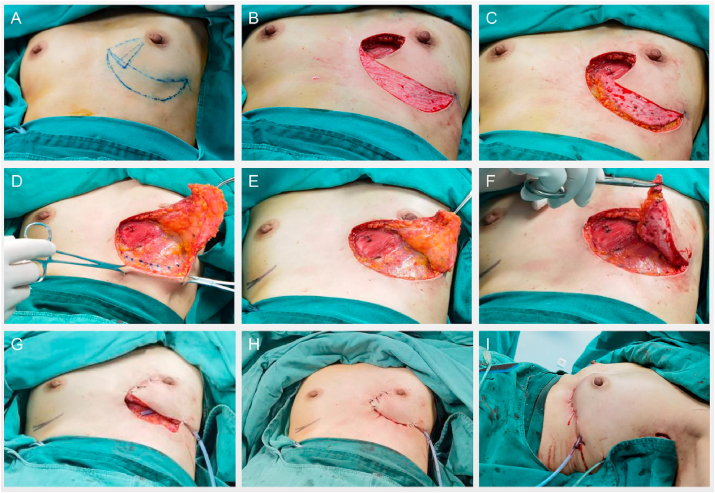
The step by step operation of Zhuo's technique in clinical case.

Nine patients with breast cancer in the LIQ received Zhuo's oncoplastic technique, between January 2017 and August 2019. The patients' baseline characteristics are shown in Table 1. The median age of the participants was 54 years (range: 41–65 years). The median BMI was 24.5kg/m2 (range from 21.6 to 30.0 kg/m2), and had varying cup sizes: one patient had C cups (11.11%), two patients had A cups (22.22%), all the other six patients had B cups (66.67%). Most tumors were identified as Luminal B subtype (7/9) and invasive ductal carcinoma (IDC) (8/9), and one was a triple negative breast cancer (TNBC). The median histological size of lesion and clinical size were 18.0 mm (range: 12–35 mm) and 25.0 mm (range: 15–40 mm), respectively. SLN biopsy results for all patients were negative. No patients presented axillary lymph nodes metastases. Fig. 2 shows the step by step tumor resection provided to all patients using the Zhuo's technique.

**Table 1:** Baseline character of patients.

Cases ID	Age (years)	Cup	BMI (kg/m^2^)	Diabetes	Subtype	Histology	Clinical Tumor Size (mm)	Pathology Tumor Size (mm)
1	52	B	24.9	No	Luminal B	IDC	25	18
2	54	B	28.0	No	Luminal B	IDC	30	25
3	51	B	24.0	No	Luminal B	IDC	30	25
4	51	C	30.0	No	Luminal B	IDC	25	18
5	45	A	22.6	No	TNBC	IDC	20	15
6	65	B	21.6	No	Luminal B	IDC	15	12
7	54	B	24.5	No	Luminal A	IDC	15	12
8	62	A	23.5	No	Luminal B	IDC	25	20
9	63	B	24.5	No	Luminal B	MA	40	35
Mean	54	/	24.5	/	/	/	25	18

BMI: Body Mass Index; TNBC: Triple negative breast cancer; IDC: Invasive ductal Carcinoma.

MA:Mucinous adenocarcinoma.

The specific information regarding surgery and follow-up is presented in Table 2. Eight patients achieved negative resection margins at the first rapid freezing. One patient with a positive margin at first tumor excision underwent an enlarged excision until the margin was reported negative, resulting in a big defect; therefore, it was not possible to offer her the II phase reconstruction procedure. The median operation time was 90.0 min (range: 70–130 min), including the time for frozen biopsy. The median intraoperative blood loss was 50 ml (range: 30–100 ml). Postoperative complications, such as bleeding, fat liquefaction, and flap necrosis, were not observed.

**Table 2:** Surgery information and follow up of patients.

Cases ID	Operation time (min)	Re-excision	Bleeding-out (ml)	Complications	Cosmetic results	Therapy	Follow-up (months)	Recurrence status
1	130	Yes	100	No	4	C + R + E	41.5	0
2	80	No	50	No	5	C + R + E	39.5	0
3	115	No	30	No	5	C + R + E	37.5	0
4	90	No	50	No	5	C + R + E	34.0	0
5	70	No	30	No	5	C + R	27.0	0
6	80	No	40	No	5	C + R + E	20.5	0
7	100	No	50	No	4	C + R + E	19.0	0
8	80	No	30	No	5	R + E	18.0	0
9	110	No	80	No	5	C + R + E	17.5	0
Mean	90	/	50	/	5	/	27	/

C: Chemotherapy; R: Radiation therapy; E: Endocrine therapy.

**Fig. 3:**
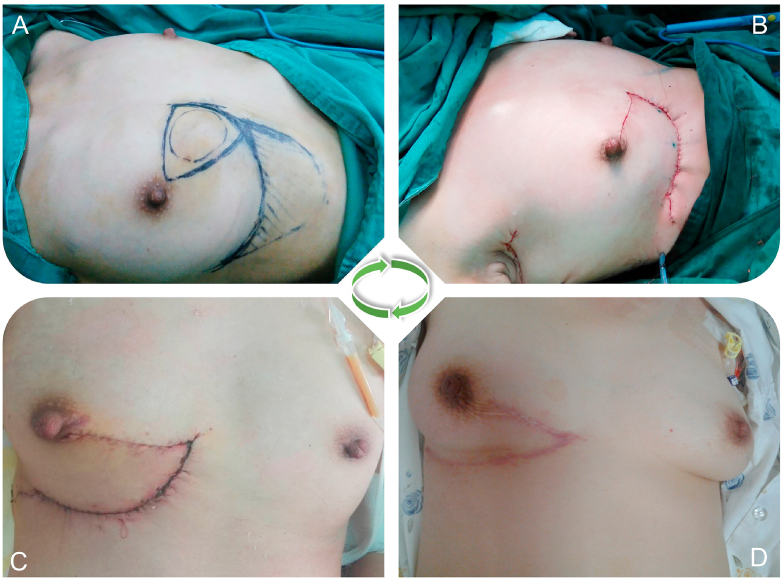
Aesthetic outcome of one patient who receive Zhuo's technique, including preoperative(A), intra operative(B), aspect of one month(C) and six month after surgery(D).

The median follow-up time was 27.0 months (range: 17.5–41.5 months) and none of the patients had local recurrences or metastases. All patients with the subtype of luminal A or B received radiotherapy, chemotherapy and/or endocrine therapy. One patient stopped chemotherapy due to intolerable side-effects and received a combination of radiotherapy and continued endocrine therapy (Table 2). Cosmetic evaluation was available for all patients. Seven patients (77.8%) achieved an aesthetic score of 5 and two patients (22.2%) achieved a 4 point score. Fig. 3 shows an exemplary aesthetic outcome of one patient who received Zhuo's technique, including preoperative (Fig. 3A), intraoperative (Fig. 3B) and follow-up after one (Fig. 3C) and six months (Fig. 3D).

## 4 Discussion

OPBS technology can expand the indications of traditional reconstructive BCS, but not all patients can obtain satisfactory aesthetic results. This is because tumor location, breast size, and the relative ratio of the resected tissue to the breast volume may affect cosmetic outcomes [10,11]. Compared with other quadrants, the rotation of breast tissue in LIQ is more difficult. This is even more so the case in patients with small to medium breast volume. Thus, performing OPBS for tumors located in the LIQ of small to medium volume breasts is a challenge for reconstructive breast surgeons.

In this study, we present the results of a novel and safe OPBS technique for women with small to medium breast volumes who present with breast cancer in LIQ, including a novel incision shape for tumor excision and the use of an abdominal flap for surgical defect repair. In the present study, the enrolled patients mainly had small to medium breast volume (B cup: 66.67%; A cup: 22.22%; C cup: 11.11%), medium size tumors (range: 15–40 mm; median 25.0 mm). These results suggests that our new technique could be applied to international patients with small to medium sized breasts, thus extending the benefits of this safe and satisfying reconstructive technique to more patients.

A suitable incision can provide the surgeon with operative convenience while ensuring the complete resection of the tumor and positively increasing the postoperative satisfaction of the patient. Other techniques are used frequently for defect reconstruction. Lee et al. [12] designed a fish-hook-shaped skin (FHS) incision from an axillary site to the tumor for patients with cancer in the inner and lower quadrants. However, unlike the incision we designed, the FHS is mainly filled with a superior-based dermoglandular tissue flap above the tumor, while we use the pedicle flap from the upper-abdomen. In addition, the incision scar after FHS incision is longer when compared with our incision. Another option is the use of thoracoepigastric flap (TEF), in which the blood supply comes from superior epigastric perforator artery and the incision is designed from the lower lateral angle of the defect and then curves laterally down along the midaxillary line [13]. When compared with our technique, both techniques use abdomen flaps to repair defects and both methods can provide enough tissue to cover the defect through flexible incision design. However, the most significant difference between the two techniques is the indication of use. Zhuo's technique is mainly recommended for inner quadrant defect repair and TEF technique is usually used for partial defect repair after mastectomy. In addition, similarly disadvantaged to FHS, TEF also leaves a long upper-abdomen scar while Zhuo's leave a smaller scar.

In 2012, Clough [9] developed the LIQ-V technique to provide a convenient method for removing tumors in LIQ, consisting of a simple V-shaped incision. Patients with large size breasts who undergo LIQ-V technique obtain good aesthetic outcomes. For small to medium breasts with limited volume, this approach can lead to a larger defect, even if the tumor is small. Hence, it complicates or even hinders the rotation of the glandular tissue; therefore, this method is not applicable for patients with small to medium breast volume. On the contrary, the Zhuo's technique uses a crescent-shaped incision ensuring that the tumor is completely removed and the surgical defect is easily covered by the abdominal flap. The IMF is the upper edge of our incision, which can be inverted with the lower edge of the incision in the later repair. After the reconstituted new IMF is healed, the scar will not be visible when the patient is standing. This can greatly improve the cosmetic appearance of the breast.

When compared to those with large size breasts, patients with small to medium sized breasts usually lack sufficient glandular tissue for filling the defect after tumor excision. Therefore, flaps are a better choice for solving this problem. Similarly to our starting point, Yuko Kijima [14] developed a technique which uses the inframammary adipo-fascial flap to fill the defect in the LIQ after breast-conserving surgery. This technique provides a positive reference for solving the problem of insufficient filling of the middle and lower breast tissues. Moreover, lateral intercostal perforators (LICAP) can be used to reconstruct defects in the outer quadrant of breasts with small and medium volume; however, it is not appropriate for reconstructing defects in the inner quadrant because the lateral intercostal artery vascular pedicle is relatively short. In contrast, our pedicle flap can be easily used to eliminate the surgical cavity after folding. Because our pedicle is wide enough, the blood supply to the flap can be effectively guaranteed, which can prevent parenchymal undermining and necrosis. More importantly, this simple and convenient technique does not require professional plastic surgery training, and can be performed by breast surgeons who are proficient in simple glandular tissue reshaping techniques.

Furthermore, operation-related complications can affect postoperative radiotherapy and chemotherapy. In our study, no postoperative complications were observed in patients, nor were their adjuvant treatments delayed. Studies have demonstrated that OPBS does not increase the recurrence rate of breast cancer when compared with BCS [15]. The preliminary results of this study also support this conclusion.

Aesthetic results are an important evaluation index for OPBS technique. Compared with traditional inverted T solutions, Zhuo's technique effectively avoids “break” deformities, thereby providing patients with improved aesthetic outcomes. In addition, when glandular tissue is rotated to the inner quadrant, it will inevitably bring about NAC malpositioning. This is caused by the limited range of the glandular tissue's activity. Our technique can better reduce the probability of this event, as capacity is freed in the retromammary space, which greatly improves the mobility of the glandular tissue during rotation. The results of our aesthetic evaluation also support this conclusion, as most of our patients achieved high aesthetic scores of 4 or 5-points. Moreover, considering the importance of postsurgical symmetry with the contralateral breast, this achievement could affect the aesthetic outcomes [16]. Zhuo-technique uses the IMF of the healthy side as a reference from which to reconstruct a new, symmetrical IMF on the affected side, which may also reduce the risk of NAC mal-positioning. This difference could also explain the good aesthetic outcomes of the proposed technique.

As reported for other OPBS techniques [17], we do not recommend the Zhuo-technique for patients with uncontrolled diabetes, history of previously irradiated breasts, tobacco addiction and other factors which are known to affect the healing of the incision. In addition, although the Zhuo's technique has advantages for patients with small sized breasts (excisions encompassing 20%–50% of breast volume), those with excisions more than 50% of volume, may not be suitable for breast reconstruction with this surgical approach.

### Strengths and limitations of the study

This is the first time that an analysis of the Zhuo-technique has been carried out. Similar to other pilot retrospective studies, this study has some limitations as a result of small data availability. Nevertheless, some patients who underwent Zhuo's technique were followed up on for more than three years and no local recurrence or distant metastases occurred. These results suggest that the Zhuo-technique is oncologically safe and effective, as well adds to the international knowledge on the clinical outcomes of OPBS. However, long-term oncological outcomes should be evaluated in further comparative studies.

## 5 Conclusions

Insufficient glandular tissue in the lower quadrant of the breast is the main reason for the difficulty in repairing defects after oncoplastic surgery. Specially after resection of tumors in the lower inner quadrant, Zhuo's oncoplastic technique could provide an advantageous and flexible surgical approach allowing sufficient glandular tissue transposition for the reconstruction of the defect created in small to medium volume breast. Finally, this new approach allows a satisfactory symmetry with the contralateral breast*.* We encourage other surgical working groups to investigate the middle- and long-term effects of this new technique.

## Ethical Approval

The protocol was approved by the Institutional Review Ethics Committee(GTCMH-2020-100; 2020.09.01). The study was conducted according to the Declaration of Helsinki principles

## Research Registration Unique Identifying Number (UIN)

Chinese clinical Trial Registry Nr: ChiCTR2100043484

Available from URL: http://www.chictr.org.cn/showproj.aspx?proj=122030

## Source of funding

This study was partially supported by the University Medicine Oldenburg, Carl von Ossietzky University Oldenburg.

## Authors’ contributions

RDW and RZ: Conceptualization. WS and LATR: Data collection, formal analysis. WS, LATR; JD, JZ, YJ: writing first report. WS and LATR: Draw demo figure. All authors read and approved the final manuscript.

## Ethics approval and consent to participate

Approval of the local ethics committee was obtained before data collection and analysis for this retrospective study.

## Guarantor

Dr. Rui Zhuo, Department of Breast Surgery, Gui Lin TCM Hospital of China, Guilin, 541002, Guang Xi, P.R China. E-mail: drzhuorui@hotmail.com

## Provenance and peer review

Not commissioned, externally peer-reviewed.

## Declaration of competing interest

The authors declare that they have no competing interests.
